# Mapping relational links between motor imagery, action observation, action-related language, and action execution

**DOI:** 10.3389/fnhum.2022.984053

**Published:** 2022-11-04

**Authors:** Helen O’Shea

**Affiliations:** Department of Psychology, University of Limerick, Limerick, Ireland

**Keywords:** motor cognition, motor imagery, action observation, covert action, simulation, embodied, multidimensional model, action-related langauge

## Abstract

Actions can be physically executed, observed, imagined, or simply thought about. Unifying mental processes, such as simulation, emulation, or predictive processing, are thought to underlie different action types, whether they are mental states, as in the case of motor imagery and action observation, or involve physical execution. While overlapping brain activity is typically observed across different actions which indicates commonalities, research interest is also concerned with investigating the distinct functional components of these action types. Unfortunately, untangling subtleties associated with the neurocognitive bases of different action types is a complex endeavour due to the high dimensional nature of their neural substrate (e.g., any action process is likely to activate multiple brain regions thereby having multiple dimensions to consider when comparing across them). This has impeded progress in action-related theorising and application. The present study addresses this challenge by using the novel approach of multidimensional modeling to reduce the high-dimensional neural substrate of four action-related behaviours (motor imagery, action observation, action-related language, and action execution), find the least number of dimensions that distinguish or relate these action types, and characterise their neurocognitive relational links. Data for the model comprised brain activations for action types from whole-brain analyses reported in 53 published articles. Eighty-two dimensions (i.e., 82 brain regions) for the action types were reduced to a three-dimensional model, that mapped action types in ordination space where the greater the distance between the action types, the more dissimilar they are. A series of one-way ANOVAs and *post-hoc* comparisons performed on the mean coordinates for each action type in the model showed that across all action types, action execution and concurrent action observation (AO)-motor imagery (MI) were most neurocognitively similar, while action execution and AO were most dissimilar. Most action types were similar on at least one neurocognitive dimension, the exception to this being action-related language. The import of the findings are discussed in terms of future research and implications for application.

## Introduction

Motor cognition relates to any mental process that is associated with neural activity in the motor system (Jeannerod, 2006). It concerns mental functions such as action planning, motor imagery, action observation, and action-related language, which are assumed to be interconnected through their reliance on neural sensorimotor systems (Grèzes and Decety, 2001; Jeannerod, 2001; Prinz, 2014). In particular, motor imagery (MI) is a dynamic process whereby actions are mentally generated and unfold over time without physical movement execution (Decety, 1996; Jeannerod, 2006). Similarly, action observation (AO) involves implicit dynamic mental processes that represent action content and are triggered when observing other’s actions (Jeannerod, 2006; Rizzolatti and Sinigaglia, 2010). The aim of this study is to explore the interconnectedness between different action processes using non-metric multidimensional scaling (NMDS). This technique involves mapping dis/similarities between psychological entities (which in this study are different action types) in a multidimensional space, where the distances between entities indicate the extent of relatedness between them (Shepard, 1962; Hout et al., 2013).

Covert (or motor-cognitive) actions, such as MI or AO, are deemed similar to physical action execution because all supposedly have access to overlapping and interacting motor and cognitive systems (Ramsey et al., 2021). The latter cognitive system includes the component processes involved in mental action representation, such as attention, memory, and inhibitory control processes which facilitate, for example, action goal and plan formation (see O’Shea and Moran, 2017). It is generally recognised (with substantial empirical support) that covert actions allow us to activate neural motor systems offline (i.e., cognitively through internal representational processes and without physically moving; e.g., Hardwick et al., 2018; Courson and Tremblay, 2020), thus offering the prospect of improving or rehabilitating the functioning of these systems (Caligiore et al., 2017; Paravlic, 2022), inducing neuroplastic change (e.g., Baeck et al., 2012; Debarnot et al., 2014), learning new skills (e.g., Kraeutner et al., 2016; Ingram et al., 2019), and/or improving behavioural skill (e.g., Schuster et al., 2011; Di Rienzo et al., 2016). It is proposed that action information and effect information are merged in internal motor representations (i.e., mental knowledge structures), and the motor system is primed and the sensory consequences of action anticipated (Wolpert and Ghahramani, 2000; Kilintari et al., 2014; Kilteni et al., 2018). Research in this field has largely focused on exploring the similarities between various covert actions and action execution with the aims of: (i) using covert actions, such as MI, as an adjunct to physical action to improve movement skill or develop movement rehabilitation techniques (e.g., Di Rienzo et al., 2016; Bek et al., 2021); (ii) elucidating the neurocognitive architecture of human movement control (Schack and Ritter, 2009; Land et al., 2013; Rosenbaum, 2021); and (iii) developing a unified theoretical framework accounting for the interconnections between perception, cognition, and action (Wolpert and Ghahramani, 2000; Hommel et al., 2001; Jeannerod, 2001; Hommel, 2019).

One widely explored theoretical framework accounting for similarities between different action types is motor simulation theory (MST; Jeannerod, 2001, 2006; for review see O’Shea and Moran, 2017), which suggests that covert actions (i.e., MI, AO, perceptually-based decisions, action-related language, etc.) and physical action execution utilise similar motor representational processes (Jeannerod, 2001, 2006). In this regard, all action types (e.g., physical execution, MI, AO, action-related language) assemble mental motor representations of the intention to act, which contain action plans and motor programs that guide subsequent physical action execution or, as in the case of MI for example, the mental unfolding or simulation of the action (Jeannerod, 1994, 2006). Shared mental representational processes signify that different mental and physical action types are alternative ways of generating an action, and so, can be considered functionally equivalent (Jeannerod, 1994, 2006; although see Glover and Baran, 2017). Additionally, mental motor simulation processes are also assumed to provide pragmatic knowledge of action-related words during semantic language processing, and therefore assist in meaning extraction and learning (Jeannerod, 2006; Bonnet et al., 2022). Overlapping motor and cognitive systems, whereby cognitive action states (e.g., MI or action-related language) activate motor centres in the brain, is a key advantage for human behaviour because it offers opportunities for behaviour rehabilitation or improvement, wherein one action type is used to enhance another when dysfunction occurs.

The idea of interconnected motor-cognitive systems is captured not only by MST (Jeannerod, 2001, 2006), but also by several other prominent theoretical views. In this regard, several potential mechanisms have independently been proposed to drive perceptual-cognitive-motor processes, including association (Barsalou, 1999, 2008; Hesslow, 2012; Pulvermüller, 2018), emulation (Grush, 2004), simulation (Barsalou, 1999, 2008; Jeannerod, 2001, 2006), perception-action coding (Hommel et al., 2001; Hommel, 2019), or predictive process (Wolpert and Ghahramani, 2000) mechanisms. These theoretical views differ somewhat in their central tenets and mechanisms (of which an in-depth discussion is beyond the remit of the present study, and so the reader is encouraged to explore the cited authors in relation to the details of each) but can be considered broadly compatible because they highlight links between action and the effects of that action, and suggest that lower-level sensorimotor systems are necessarily integrated into larger cognitive systems, that facilitate action planning and programming processes, anticipation of action and sensory outcomes, behaviour mimicking, conceptual processing, and so on. In relation to the nature of the functional interconnections between perceptual, cognitive, and motor systems, whether the aforementioned mechanisms promote automatic association (the result of learning processes; Pulvermüller, 2005, 2012; Hesslow, 2012) or representational and simulation processes (the dynamic assembly and anticipatory processing of sensorimotor information; Jeannerod, 2001; Grush, 2004) may have subtly different manifestations in any overlap between the systems. It might be expected that interconnections between motor, perceptual, and cognitive systems *via* (Hebbian-like) association would be tighter and/or more complete across motor and cognitive functions than those based on simulation or emulation processes. To provide an example, when the cognitive system is engaged in action-related language processing, association mechanisms predict that sensorimotor systems are cognitively (re)used in an implicit manner to extract word meaning (Pulvermüller, 2018). This mechanism is a consequence of learning where action-related words and their associated neurocognitive sensorimotor functions are automatically and computationally interlinked (i.e., Barsalou, 1999; Hauk et al., 2004; Pulvermüller, 2012, 2018). A consequence of such a close interconnection would be that action performance could facilitate action-related language processing, and* vice versa*. Alternatively, simulation and emulation mechanisms appear to operate on (at least partially) identified semantic information, in that, the action-related linguistic information is simulated in motor systems after initial meaning-related processing so that a pragmatic knowledge of the word is achieved (Jeannerod, 2006). Different theoretical accounts of motor-cognitive processes highlight the need for continued investigation into the nature of the interconnections between motor and cognitive systems, because developing a single overarching theoretical framework will ultimately benefit application in this area (e.g., how best to exploit interconnections between different systems in the rehabilitation of any one that is dysfunctional).

If motor representational, simulation, or association processes support the covert action processes of MI, AO, and action-related language, it can be expected that activity in their underlying neural systems will overlap due to a shared fundamental neurocognitive mechanism driving their functioning; this is largely what has been reported, albeit across different combinations of action types (Hétu et al., 2013; Hardwick et al., 2018; Courson and Tremblay, 2020). Shared neuroanatomical structures have been demonstrated for AO, MI, and action execution (Caspers et al., 2010; Hétu et al., 2013; Hardwick et al., 2018). In this regard, action execution has been found to share a more similar and/or extended network with MI than with AO (Hardwick et al., 2018; Courson and Tremblay, 2020). Overlapping brain regions between MI and execution typically include the supplementary motor area (SMA), premotor cortex (PMC), posterior parietal areas (PPC; including inferior and superior parietal lobes, IPL, SPL), basal ganglia, and cerebellum (Hardwick et al., 2018). AO also activates the SMA, PMC, parietal, and additionally the occipital areas, but typically fails to activate subcortical areas, such as the cerebellum (Hardwick et al., 2018; although see Calvo-Merino et al., 2006). Some areas are suggested to be distinct to MI, for example, the dlPFC, and given the absolute reliance of MI on stored information and internal manipulation of such information, it is not surprising that this area, which is associated with active or working memory, is triggered (e.g., Frith and Dolan, 1996; Hardwick et al., 2018). Regarding action-related language, there is evidence of activity in neural motor centres during language processing (for review see Jirak et al., 2010; Yang and Shu, 2014; Courson and Tremblay, 2020), and this appears to be somatotopically organised (Hauk et al., 2004; Pulvermüller, 2005). Furthermore, research demonstrates overlap in the frontoparietal neural circuits supporting action-related language and those supporting action execution (e.g., Harpaintner et al., 2020), mental representation of action (Péran et al., 2010), and AO (Meister and Iacoboni, 2007; Courson and Tremblay, 2020).

Challenging the idea that different action-types are similar in nature, are recent theoretical proposals of “dual-simulation” where, for example, AO and MI can hypothetically be simultaneously represented in the brain and recent research findings relating to the effects of various combinations of action types (Vogt et al., 2013; Eaves et al., 2016; Bruton et al., 2020). For instance, empirical evidence demonstrates facilitation of corticospinal excitability (CSE; an indication of activity in primary neural motor centres) when individuals simultaneously observe and imagine hand actions, which is greater than that found during AO alone (Wright et al., 2014; Cengiz et al., 2018; Kaneko et al., 2018; Bruton et al., 2020). The facilitation observed may be driven by MI rather than by the AO component of the combination, because evidence indicates that whether the imagined hand movement uses the same or different fingers to those observed, it is the imagined effector (and not that used in the observed action) that produces the facilitation effect (Meers et al., 2020). AO alone compared to baseline did not influence CSE which indicates some dissimilarity between AO and MI (Kaneko et al., 2018; Meers et al., 2020). Additionally, while early learning of coordinative and sequential actions can occur using AO (Boutin et al., 2010; Gonzalez-Rosa et al., 2015; Cuenca-Martínez et al., 2020), learning has been shown to be influenced by MI ability, in that, individuals with high imagery ability have been seen to perform significantly better following observational learning than those with low imagery ability (Lawrence et al., 2013). So, performance improvements following concurrent AO-MI use may be driven by the MI component. Notwithstanding this, practicing an action using simultaneous MI and AO can lead to better outcomes than either alone (see Scott et al., 2021; Bruton et al., 2020), and so, different action types may have unique processing components that allow them to complement each other in achieving positive effects (Vogt et al., 2013; Eaves et al., 2016). Extrapolating from the idea that there may be some level of uniqueness between action types, it might be that the existence of somewhat distinct but complementary processes also explains why training using physical practice interspersed with AO (Larssen et al., 2021; Bazzini et al., 2022) or MI (e.g., Allami et al., 2008; Malouin et al., 2013; Rozand et al., 2016) achieves better performance or movement retention outcomes than using physical practice alone. In a similar manner, behavioural evidence shows that combining action-related language with MI (Bonnet et al., 2022) or action execution (Larson and Suchy, 2015), improves performance on language comprehension tasks and motor sequence learning and control, respectively.

In support of some level of uniqueness across action types, research indicates that they may be dissociable in terms of the neural representations underlying their functioning (Lui et al., 2008; Péran et al., 2010; Macuga and Frey, 2012; Vry et al., 2012; Wang et al., 2022). In this regard, distinct neural signatures are evident across action types (Gerardin et al., 2000; Vry et al., 2012; Hardwick et al., 2018), with the amount and pattern of activation seemingly hierarchically organised, for example, in sensorimotor, SMA, and cerebellum regions, action execution displays the greatest increase in activity over resting baseline, followed by MI, and finally AO (Macuga and Frey, 2012). Further, and perhaps not surprisingly, covert action is typically associated with weaker neural activity than action execution (Sharma and Baron, 2013; Avanzino et al., 2015). Evidence relating to the neural substrate of language also indicates a motor gradation across different action types (see Courson and Tremblay, 2020). Specifically, discrepancies are evident in the shared network between mental representation of action and action-related language (Péran et al., 2010; Yang and Shu, 2014), with for example, activity in the overlapping frontoparietal network showing more involvement of parietal areas during action mental representation whereas action-related language appears more supported by frontal areas (Péran et al., 2010). Further, MI of hand-related verbs has been found to activate the SMA, an area important for the planning and sequencing of voluntary movement, more strongly than reading the same verbs (Cunnington et al., 2005; Yang and Shu, 2014).

In summary, despite accumulated evidence supporting the idea that cognitive and motor systems are elaborately interconnected, no single explanatory framework exists that fully captures the extent and/or nature of these interconnections. This is likely due, firstly, to the complexities associated with studying covert action processes, whereby overt behaviour is discouraged and so resourceful techniques and approaches are required to gain insight into different covert actions, and secondly, because the neurocognitive substrate of different action types is characteristically multidimensional, involving a large number of neuroanatomical locations and connections associated with information processing (Young et al., 1995). Given this, the primary aim of the present study is to reduce the high-dimensional neural substrate of four action-related behaviours to characterise and further elucidate the neurocognitive connections between them. To achieve this aim, a methodological approach that is novel in this field was adopted, non-metric multidimensional scaling, to transform the high-dimensional data so that they are projected onto fewer dimensions, that are graphically displayed and make any intrinsic patterns between the four action types more apparent (for a more comprehensive description of this method, see “Materials and method” section below; Pielou, 1984).

Based on existing literature, a number of predictions were made: (i) motor-cognitive simulation processes are covert and involve little or no physical action execution, and so, it is expected that some dissimilarity will be observed, as indicated by the distance between data points in ordination space on at least one dimension, between covert actions (i.e., MI, AO, and action-related language) and action execution; (ii) empirical evidence shows behavioural and neurophysiological overlap between MI and action execution (e.g., Di Rienzo et al., 2016), and so, it is expected that less dissimilarity will be observed between action execution and MI on at least one dimension, than between action execution and the covert action types of AO and action-related language; (iii) although AO promotes early learning of unfamiliar or novel actions (e.g., Gonzalez-Rosa et al., 2015), it is typically considered a perceptual-cognitive function (see Kim et al., 2017) with less neural activation in purely motor centres than other mental action types (e.g., Caspers et al., 2010; Hardwick et al., 2018), and so, it is expected that AO will be most dissimilar to action execution across dimensions; and finally; and (iv) given that links between action-related language and physical execution (e.g., Harpaintner et al., 2020) and AO (Meister and Iacoboni, 2007) have been independently found to positively influence language processing (i.e., Beauprez et al., 2020; Courson and Tremblay, 2020), it is expected that the data points in the dimensional space associated with action execution and AO will show some proximity to those of action-related language on at least one dimension. No predictions were formed in relation to combined AO-MI because it is a relatively new area for neurophysiological study. It seems likely however, that combined AO-MI will be proximal to MI and/or AO in ordination space on some dimension, thus representing similarity between the action types. Additionally, it could be anticipated that because action execution involves both perceptual (similar to AO) and predictive (similar to MI) processes for control of movement in the environment (e.g., Wolpert and Ghahramani, 2000; Grush, 2004; Pezzulo et al., 2013; Deschrijver et al., 2017) there will be some proximity on at least one dimension between action execution and combined AO-MI.

## Materials and Method

### Literature search

PubMed database^1^ literature searches for neuroimaging articles were performed over 1 month (January 2022; up-dated in May 2022). Combined searches of terms relating to different action types and neuroimaging methods provided data for multidimensional modeling of dissimilarities between different covert action types and action execution processes. The primary search used the string (“motor imagery” OR “action observation” OR “action language” OR “verb”) AND (“fMRI” OR “neuroimaging” OR “PET”) NOT (“clinical” OR “children” OR “stroke” OR “review” OR “monkey” OR “MEG” OR “fNIRS” OR “neurofeedback” OR “primate” OR “BCI”), which returned 690 results (212 MI related; 233 AO related; 245 language related). Subsequent searches were more targeted towards AO+MI and language-related articles and used variations of this search string (e.g., “motor imagery” AND “action observation”, or “action” AND “language” AND “fMRI”) which returned 15 and 147 results, respectively. All articles (*N* = 852) were initially screened using information in the title and/or abstract (requiring information relating to action types and neuroimaging). Reference sections in reviewed articles and known articles (not returned in searches) were also screened for additional relevant studies. If action execution was used as a second or control condition in a study of a different action type it was included as a case of action execution in the non-metric multidimensional scaling analysis. The objective here was to limit any confounding effects associated with heterogeneity in covert and overt action tasks across studies (e.g., where differences in neural activation might be linked to experimental task). In the final selection of included studies, the studies that investigated more than one action type with the same task/movement protocol across actions were prioritised over those only studying one action type.

### Inclusion—exclusion criteria

Of the 852 articles identified, those assessed for eligibility for inclusion in the final analysis were required to satisfy strict inclusion criteria, which were: (i) only studies performing whole brain analyses (not region of interest studies, as a focus on certain brain regions may bias the final model) with reporting of active regions including coordinates in standard stereotaxic space, such as MNI/Talairach, or clearly defined Brodmann areas; (ii) healthy adult participants; (iii) MI was “pure” MI in a kinaesthetic modality (i.e., not visual in nature); (iv) AO-MI was concurrent AO and MI as evidence suggests that this type is the most effective and used form for positive behavioural outcomes (e.g., sequential or coordinative AO-MI were not included; see Vogt et al., 2013); (v) articles were in English language; and (vi) neural activity during covert actions was compared to a rest or baseline state or a control task that was consistent across actions/conditions. In the selected articles, data extracted were statistically significant active brain areas during MI, AO, action-related language, and/or action execution. Note that laterality was not included as a variable in the dataset in sensorimotor-specific related areas as lateral brain activity can be strongly task and/or limb related.

The search led to 129 articles that fully met the inclusion criteria (38 AO; 33 MI; 24 action execution; 10 AO+MI; 24 action-related language). From these articles, a maximum of 100 articles was desired for input to the final multidimensional model. So, all articles investigating more than one action type were included first (minimising heterogeneity), and once this was achieved, any deficit in count was made up by randomly selecting from the remaining articles (to reach the desired 22 articles per action type). The only caveat to this random selection process was that if a meta-analysis existed among the remaining articles this was automatically included. The final dataset comprised 98 cases, that is, 98 covert action types (22 AO; 22 MI; 22 action execution; 22 action-related language; and 10 AO + MI) as studied and published across 53 scientific journal articles (for list of articles, see Table 1; note, each action type was examined for activity in 82 brain regions; for regions, see Table 2).

**Table 1:** Published articles by author name included in the final multidimensional model.

**Author, Year**
1. Baumann et al. (2022) 2. Baumgaertner et al. (2007) 3. Berlingeri et al. (2008) 4. Beudel et al. (2011) 5. Brihmat et al. (2018) 6. Casiraghi et al. (2019) 7. Caspers et al. (2010) 8. Cross et al. (2006) 9. De Grauwe et al. (2014) 10. de Vega et al. (2014) 11. Desai et al. (2013) 12. Elli et al. (2019) 13. Garbin et al. (2012) 14. Gerardin et al. (2000) 15. Grèzes and Decety (2001) 16. Hanakawa et al. (2003) 17. Hardwick et al. (2018) 18. Hauk et al. (2004) 19. Hernández et al. (2014) 20. Hétu et al. (2013) 21. Higuchi et al. (2012) 22. Iseki et al. (2008) 23. Jirak et al. (2010) 24. Kilintari et al. (2016) 25. Kuhnke et al. (2020) 26. Kuhtz-Buschbeck et al. (2003) 27. Lindenberg et al. (2012) 28. Lui et al. (2008) 29. Macuga and Frey (2012) 30. Mouthon et al. (2018) 31. Nedelko et al. (2010) 32. Nedelko et al. (2012) 33. Péran et al. (2010) 34. Plata Bello et al. (2014) 35. Popp et al. (2019) 36. Rizzolatti et al. (1996) 37. Rousseau et al. (2021) 38. Saiote et al. (2016) 39. Sauvage et al. (2013) 40. Savaki et al. (2022) 41. Schuil et al. (2013) 42. Simos et al. (2017) 43. Taube et al. (2015) 44. Tian et al. (2020) 45. Tremblay and Small (2011) 46. Villiger et al. (2013) 47. Vry et al. (2012) 48. Wang et al. (2022) 49. Willems et al. (2010) 50. Yang and Shu (2014) 51. Yang et al. (2011) 52. Zapparoli et al. (2013) 53. Zhang et al. (2017)

**Table 2:** Brain regions examined for activity during all action types (brain regions were identified according to their label and/or their Brodmann area, as reported in the articles).

**Brain Region**	
1. Precentral gyrus 2. Rolandic operculum 3. Pre-supplemental motor area 4. Supplementary motor area 5. Caudal Supplementary motor area 6. Superior frontal gyrus 7. Cingulate cortex 8. Cingulate sulcus 9. Cingulate sulcus, motor area 10. Inferior frontal gyrus 11. Left Inferior frontal gyrus 12. Right Inferior frontal gyrus 13. Inferior frontal gyrus pars triangularis 14. Right IFG pars triangularis 15. Leftt Inferior frontal gyrus pars triangularis 16. Inferior frontal gyrus pars opercularis 17. Right Inferior frontal gyrus pars opercularis 18. Left Inferior frontal gyrus pars opercularis 19. Inferior frontal gyrus pars orbitalis 20. Right Inferior frontal gyrus pars orbitalis 21. Left Inferior frontal gyrus pars orbitalis 22. Orbital gyrus 23. Frontal eye field 24. Middle frontal gyrus 25. Dorsolateral prefrontal cortex 26. Left dorsolateral prefrontal cortex 27. Right dorsolateral prefrontal cortex 28. Ventral lateral prefrontal cortex 29. Prefrontal cortex 30. Primary motor cortex 31. Central sulcus 32. Premotor cortex 33. Ventral premotor cortex 34. Dorsal premotor cortex 35. Inferior parietal lobe 36. Posterior parietal lobe 37. Superior parietal lobe 38. Precuneus 39. Intraparietal sulcus 40. Supramarginal gyrus 41. Angular gyrus 42. Superior temporal gyrus 43. Superior temporal sulcus 44. Middle temporal gyrus 45. Middle temporal sulcus 46. Inferior temporal cortex 47. Posterior temporal cortex 48. Occipitotemporal junction 49. Right fusiform gyrus 50. Left fusiform gyrus 51. Middle occipitotemporal/lingual gyrus 52. Middle temporal visual area 53. Primary visual cortex 54. Secondary visual cortex 55. Occipital cortex 56. Superior occipital gyrus 57. Middle occipital gyrus 58. Inferior occipital gyrus 59. Parieto-occipital cortex 60. Intracalcarine cortex 61. Calcarine sulcus 62. Insula 63. Right insula 64. Left insula 65. Thalamus 66. Post central gyrus 67. Primary somatosensory cortex 68. Parietal temporal operculum 69. Putamen 70. Anterior putamen 71. Posterior putamen 72. Pallidum 73. Lentiform nucleus 74. Caudate nucleus 75. Secondary somatosensory association cortex 76. Parietal operculum 77. Hippocampus 78. Cerebellum 79. Cerebellum anterior lobe 80. Cerebellum posterior lobe 81. Cerebellum flocculonodular lobe 82. Cerebellum—vermis

### Non-metric multidimensional scaling (NMDS)

Non-metric multidimensional scaling (NMDS) can be considered a method of statistical fitting that generates a spatial configuration whose distances between data points accurately reflect true dissimilarities in the data (Kruskal, 1964a,b; Spiridonov et al., 2019). The procedure involves firstly examining relationships in the data and generating a rank ordered matrix of the dissimilarities between all pairs of cases for the set of cases (each case in the present study represents a particular action type described by a number of neural attributes, i.e., brain regions that are activated or not during, e.g., MI). The goal is then to plot the data in a low-dimensional space with the minimum possible stress; with stress being a measure of how well the configuration matches the dissimilarity data (i.e., goodness-of-fit; Kruskal, 1964a). This is achieved by placing the data in either an initial arbitrary configuration or in a configuration where all data are equidistant, then determining in which direction stress is reducing most quickly and moving the data point structure in this direction (see Kruskal, 1964a,b). The procedure of determining the direction of most quickly decreasing stress (i.e., the steepest gradient) and moving the configuration in this direction is repeated as many times as necessary to arrive at a minimal stress value, and hence, an accurate spatial representation of the dissimilarities in the data. To note, unlike metric multidimensional scaling which uses absolute values of the dissimilarities in the data, NMDS only considers the rank ordering of the dissimilarities so that the distances in the final configuration are rank ordered to reflect the rank ordered data in the dissimilarity matrix (Goodhill et al., 1995). The overall aim of NMDS is to arrive at a solution or graphical/spatial model that faithfully represents the relationships between cases (i.e., action types) in a low-dimensional space so that underlying patterns (i.e., the multidimensional structure) can be identified and interpreted (for further description and uses, see Shepard, 1962; Kruskal, 1964a,b; Goodhill et al., 1995; Spiridonov et al., 2019).

The value of the NMDS modeling approach in the present study is that all neural raw data (i.e., activated neural regions in comparison to baseline/controls) across different studies and different action types can be inputted into a single model that maintains the high-dimensional status of data, but projects the dis/similarity between data in a low-dimensional space. Additionally, every dimension (i.e., neural region) associated with a specific action type in a particular study is taken into account when fitting and plotting the action type’s coordinate location relative to others in the model. Unlike other dimension reduction techniques, such as principal component analysis, NMDS can map binary data (i.e., brain areas that are activated or not) and capture dis/similarity between action types in fewer dimensions where all dimensions are visible and no dimension take priority over another.

In the present study, each action type or case is described in terms of activity across 82 brain regions (i.e., 82-dimensions) as objectively measured by scientific study. The inclusion of brain regions was based on motor control, visuomotor, and motor cognition literature. In the final dataset there were 98 action types (i.e., or cases; 22 MI, 22 AO, 22 action execution, 22 action-related language, and 10 AO-MI). Inclusion of specific brain regions was based on the motor-cognitive and motor control literature (for a list of included brain regions and studies, see Table 2). Non-metric scaling (rather than metric) was appropriate for the current data as they were at the ordinal measurement level (Shepard, 1962). Using NMDS, pairwise comparisons between all action-specific neural substrates (i.e., cases) were translated into a graphical representation in which the distances between cases reflect dissimilarity. The procedure: (i) calculates a proximity matrix of dissimilarities between all pairs of cases; (ii) rank orders these from smallest to largest; (iii) generates a set of coordinates for cases and rank orders the distances; and (iv) compares the ranked distances with the ranked proximities (Shepard, 1962). In the current analysis, data were transformed into a proximity matrix (at ordinal level; total proximities 4,753) using Lance-and-Williams Euclidean distance calculations (operating PROXSCAL in Statistical Package for the Social Sciences, SPSS 28, IBM, Chicago, IL, USA). The number of dimensions for the model was set to a minimum of two and a maximum of four. The initial configuration was Simplex, whereby all data are placed equidistant in the maximum dimension. From this, a maximum of 100 iterations (or repetitions to reduces stress and improve fit) were performed on the data. Stress plots were also generated.

## Results

### Non-metric multidimensional model

**Figure 1:**
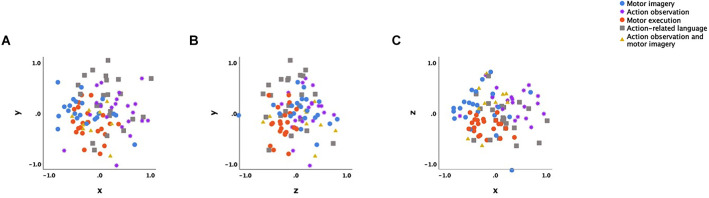
Orthogonal projection of the 3-dimensional non-metric multidimensional scaling configuration. Note: **(A)** x axis is dimension 1 and y axis is dimension 2. **(B)** z axis is dimension 3 and y axis is dimension 2. **(C)** x axis is dimension 1 and z axis is dimension 3. Each data point represents a particular action type described by neural attributes, i.e., brain regions that are activated or not. Different action types are displayed in different colours: blue, motor imagery; purple, action observation; orange, action execution; light brown, action related language; yellow, combined action observation and motor imagery.

The NMDS of action-specific brain activation produced a good fitting 3-dimensional solution (normalised raw stress = 0.03621; Tucker’s coefficient of congruence = 0.98173) after 28 iterations. Figure 1 presents the final three-dimensional NMDS ordination plot of action-specific neural substrates. The distances between data points, or action types, show how dissimilar their neurocognitive substrate are, with greater distance indicating more dissimilarity. The solution in Figure 1 is displayed as three orthogonal projections, because the three-dimensional solution resembles a cube containing the data points which is less readily interpretable.

### Statistical analysis of action type coordinates in the three-dimensional model

**Figure 2:**
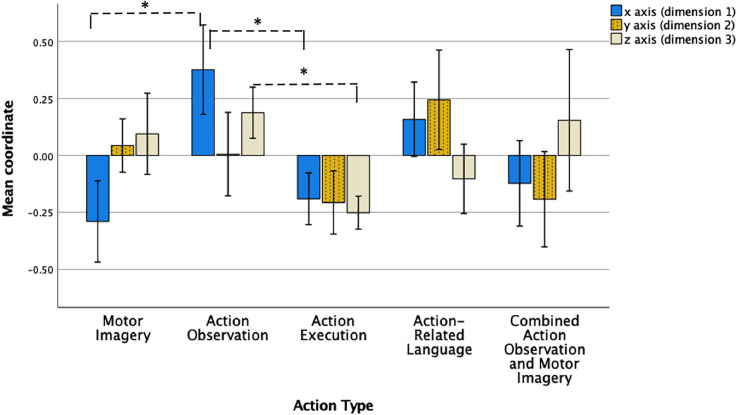
Data point coordinates across three dimensions for each action type. Note. Mean coordinates of data points for motor imagery, action observation, action execution, action-related language, and combined action observation and motor imagery across the three dimensions in the final non-metric multidimensional scaling model. Data points relating to an action type represent its neural attributes (i.e., brain regions that are activated or not). Error bars represent 95% confidence interval. * = statisitcally significant at 0.0017.

To investigate whether the differences between the locations of action type points across the three-dimensions in the final model were statistically significant, a one-way ANOVA and *post-hoc* tests were performed. Firstly, the mean coordinates of data points in ordination space for each action type on each of the three dimensions were calculated (see Table 3 and Figure 2). Descriptive analysis of these mean coordinates in the first dimension on the x axis revealed that MI, action execution, and combined AO-MI were located in a negative direction from the origin (0), while AO and action-related language were located in a positive direction from the origin (Table 3 and Figure 2). Analysis of mean coordinates in the second dimension on the y axis revealed that that MI, AO, and action-related language were located in a positive direction from the origin while action execution and AO-MI were located in a negative direction (Table 3). A final descriptive analysis of mean coordinates in the third dimension on the z axis revealed that MI, AO, and combined AO-MI were located in a positive direction from the origin while action execution and action-related language were located in a negative direction (Table 3).

**Table 3:** Mean coordinates of data points for action types across three dimensions.

	**Dimension 1 (*x* axis)**	**Dimension 2 (*y* axis)**	**Dimension 3 (*z* axis)**
**Action type**	***M* (*SD*) [95% CI]**	***M* (*SD*) [95% CI]**	***M* (*SD*) [95% CI]**
Motor imagery	−0.29 (0.40) [−0.47, −0.11]	0.04 (0.27) [−0.07, 0.16]	0.10 (0.40) [−0.08, 0.27]
Action observation	0.38 (0.44) [0.18, 0.57]	0.01 (0.41) [−0.18, 0.19]	0.19 (0.25) [0.08, 0.30]
Action execution	−0.19 (0.26) [−0.30, −0.08]	−0.21 (0.31) [−0.34, −0.07]	−0.25 (0.16) [−0.32, −0.18]
Action-related language	0.16 (0.37) [−0.004, 0.32]	0.25 (0.49) [−0.03, 0.46]	−0.10 (0.34) [−0.26, 0.05]
Combined action observation and motor imagery	−0.12 (0.26) [−0.31, 0.07]	−0.19 (0.29) [−0.40, 0.02]	0.16 (0.43) [−0.16, 0.47]

Note. CI, confidence interval; *SD*, standard deviation; *M*, mean.

While data were normally distributed in each of the dimensions (all Shapiro-Wilks tests *p* > 0.05), Levene’s test showed that the variances for action type coordinates in the second dimension on the y axis were not equal, *F*_(4,93)_ = 3.155, *p* = 0.018, and so the assumption of homogeneity of variance was not met. Accordingly, one-way independent analysis of variance (ANOVA) using the Welch *F* test (a robust test of equality of means) was used to investigate the effect of action type on the spatial configuration of coordinates in each dimension. Results revealed significant differences in mean coordinates between action types in the first dimension on the x axis, *Welch’s* *F*_(4,40.25)_ = 10.16, *p* < 0.001, the second dimension on the y axis, *Welch’s* *F*_(4,39.57)_ = 4.47, *p* = 0.004, and in the third dimension on the z axis, *Welch’s* *F*_(4,36.49)_ = 13.27, *p* < 0.001.

To investigate precisely where the differences lay, a series of pairwise comparisons of action type coordinates in each dimension were performed, using Games-Howell procedures for heterogeneity of variance. Family-wise error rate was minimised by adjusting the alpha value for the number of comparisons made, using a significance level of 0.0017. The results are presented in Table 4 and Figure 2. In the first dimension on the x axis, MI mean coordinate location (*M* = −0.29, *SD* = 0.40) was significantly different to the mean coordinate location of AO (*M* = 0.38, *SD* = 0.44), with a mean difference of −0.67, *p* < 0.001. Additionally, AO mean coordinate location (*M* = 0.38, *SD* = 0.44) was significantly different to the mean coordinate location of action execution (*M* = −0.19, *SD* = 0.26), with a mean difference of 0.57, *p* < 0.001. No significant differences were found (or survived correction procedures) in the mean coordinate locations of action types in the second dimension on the y axis. In the third dimension on the z axis, AO mean coordinate location (*M* = 0.19, *SD* = 0.25) was significantly different to the mean coordinate location of action execution (*M* = −0.25, *SD* = 0.16), with a mean difference of 0.44, *p* < 0.001.

**Table 4:** Results of pairwise comparisons of action types across three dimensions.

	**Dimension 1 (*x* axis)**		**Dimension 2 (*y* axis)**		**Dimension 3 (*z* axis)**	
**Action pair**	**M-D**	***p* (effect size)**	**M-D**	***p* (effect size)**	**M-D**	***p* (effect size)**
MI–AO	−0.67	<0.001* (1.59)	0.04	0.996 (0.09)	−0.09	0.890 (0.27)
MI–AE	−0.10	0.862 (0.30)	0.25	0.049 (0.86)	0.35	0.007 (1.15)
MI–AL	−0.45	0.003 (1.17)	−0.20	0.455 (0.53)	0.20	0.414 (0.54)
MI–AO-MI	−0.17	0.631 (0.47)	0.24	0.237 (0.83)	−0.06	0.996 (0.15)
AO–AE	0.57	<0.001* (1.58)	0.21	0.326 (0.61)	0.44	<0.001* (2.10)
AO–AL	0.22	0.401 (0.54)	−0.24	0.420 (0.53)	0.29	0.022 (0.97)
AO–AO-MI	0.50	0.004 (1.27)	0.20	0.542 (0.53)	0.03	0.999 (0.10)
AE–AL	−0.35	0.007 (1.10)	−0.45	0.007 (1.12)	−0.15	0.373 (0.57)
AE–AO-MI	−0.07	0.958 (0.27)	−0.011	1.00 (0.07)	−0.41	0.096 (1.51)
AL–AO-MI	0.28	0.132 (0.82)	0.44	0.031 (1.00)	−0.26	0.492 (0.70)

Note. Action types were MI, motor imagery; AO, action observation; AE, action execution; AL, action-related language; and AO-MI, combined action observation and motor imagery. Mean difference between pairs of mean coordinates on a dimension is significant at the alpha level = 0.0017; * = statistically significant; M-D, mean difference; *p*, alpha significance level. Pairwise comparisons were performed using Games-Howell procedures on mean coordinates; these were used for heterogeneity of variance. All action types have *n* = 22 except for AO-MI which has *n* = 10. Cohen’s *d* is reported for all effect sizes except for pairwise comparisons with AO-MI which reports Hedges’ *g* (due to unequal sample sizes).

Given the low adjusted significance value for multiple comparisons, the magnitude of the relationships between pairs of action type coordinates, in terms of dissimilarity, was examined through group-difference effect sizes (see Table 4). These can be interpreted using Cohen’s benchmarks: 0.20—small group-difference effect, 0.50—medium effect, 0.80—large effect (and Rosenthal’s 1.30 for very large effect). In the first dimension on the x axis, MI and AO, MI and action-related language, AO and action execution, AO and AO-MI, action execution and action-related language, and action-related language and AO-MI demonstrate large group-difference effects in mean coordinate location. In the second dimension on the y axis, MI and action execution, MI and AO-MI, action execution and action-related language, and action-related language and AO-MI demonstrate large group-difference effects. Finally, in the third dimension on the z axis, MI and action execution, AO and action execution, AO and action-related language, and action execution and AO-MI demonstrate large group-difference effects. These results will be considered in the “Discussion” section.

### NMDS sensitivity analysis

To identify whether the NMDS solution accurately projected the 98 cases of action types (comprising 22 MI, 22 AO, 22 action-related language, 22 action execution, and 10 AO-MI) from the proximity matrix in to the lower dimensional space, while preserving the between-cases distances from the 82 dimensions (i.e., the 82 brain regions included in the model), we examined the normalised raw stress, S-stress values, the Dispersion Accounted For, and Tucker’s coefficient of congruence.

### Dimensionality

**Figure 3:**
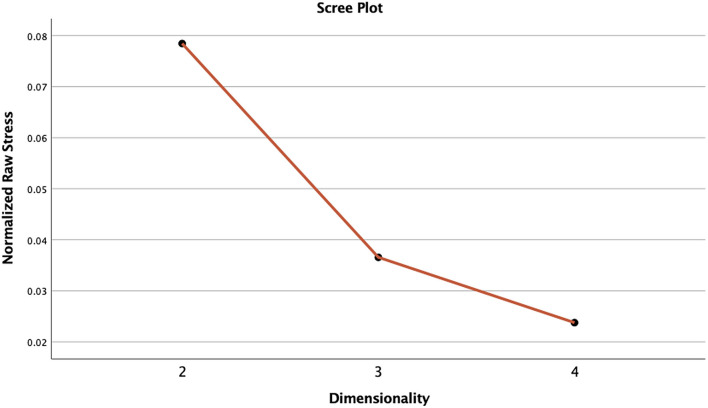
Screeplot of normalised raw stress associated with the three derived dimensional solutions. Note. The x axis displays the NMDS dimension solution for two, three, and four dimensions, and y axis displays the stress values associated with each dimensional solution, that is, how well the ranking of dissimilarities between cases correlates with the ranking of distances in the ordination structure. The elbow in the scree plot indicates the point at which there is a considerable decrease in stress which is typically assumed to indicate the best level of dimensionality for the ordination structure. In the present case, three dimensions appear to offer a good solution.

The 2-dimension NMDS solution was compared to the 3-dimension solution to assess whether the goodness-of-fit was improved and if a higher dimensional solution revealed new relationships. In this regard, the 3-dimensional model had both better fit and clearer relationship patterns (e.g., see Figure 3 for a scree plot).

### Stress

Goodness-of-fit values (e.g., stress) indicating the relationship between the ranking of ordination distances and the observed dissimilarities for both the 2-dimension solution and the 3-dimension solution were assessed in conjunction with a scree plot of normalised raw stress against dimensionality (see Figure 2). The stress value in the present study conveys the proportion of unexplained variance in the action type data by three NMDS axes (Spiridonov et al., 2019). A stress value of 0 indicates a perfect fit (i.e., no variance). The scree plot indicated an elbow at three dimensions, suggesting this is the most appropriate dimensional solution (note, an elbow is a rule of thumb indicator of the appropriate dimensional solution). Three dimensions improved the normalised raw stress by 0.04225, reducing the 2-dimension normalised raw stress of 0.07881 to the 3-dimension normalised raw stress of 0.03621. Additional measures of fit for the 3-dimenisonal solution were Dispersion Accounted For (0.96379) and Tucker’s coefficient of congruence (0.98173). A value of 1 for these measures indicates a perfect fit. Overall, the 3-dimensional solution had very good fit.

## Discussion

The aim of the present study was to characterise the interconnections between different covert and overt action types in terms of their activity in the brain (i.e., their neurocognitive relational connections). No single study has yet directly mapped the neurocognitive dis/similarity between motor imagery (MI), action observation (AO), action execution, and action-related language outside direct neurophysiological study, and so, the present study used the powerful statistical technique of non-metric multidimensional scaling (NMDS) to further elucidate the neurocognitive relational links between each of these action types. The value of NMDS modeling in this instance is that every dimension (i.e., neural region) associated with a specific action type in a particular study is taken into account when plotting the action type’s coordinate location relative to others in the model. As NMDS operates according to the idea that similarity relates to proximity (i.e., we consider items similar because they are *close* in some attribute, the colour blue might be similar to navy but dissimilar to yellow; Shepard, 1962), the relative locations of data points associated with each action type in the ordination plot were examined.

The resultant 3-dimensional model highlights some salient features regarding the intrinsic relational patterns across different action types. Specifically, the four action types of MI, AO, action execution, and action-related language were observed to cluster in four main groups across the three dimensions (Figure 1). The locations of the four clusters in the overall spatial configuration across the dimensions were found to be significantly different and so highlight dissimilarities between the various action types on three neurocognitive dimensions. Clustering indicates that each action type possesses some unique attributes in relation to their neurocognitive substrate, which is consistent with existing neurophysiological evidence (Macuga and Frey, 2012; Hardwick et al., 2018; Courson and Tremblay, 2020). However, when the average locations of each action type on each of the three dimensions were compared, only action execution and AO (in the first and third dimensions on the x and z axes, respectively) and MI and AO (in the first dimension on the x axis) showed significant dissimilarity (Table 4; Figure 2). Given the multiple comparisons performed across action types and dimensions during *post hoc* statistical analysis, effect sizes for group differences in location in the three-dimensional ordination space between pairs of action types (as calculated in the NMDS model) were used to further quantify and interpret interconnections. Large location difference, or dissimilarity, effect sizes were observed for each action type against all other action types on at least one dimension (see Table 4). For example, MI displayed large dissimilarity effects sizes (i.e., mean coordinates differed greatly in their dimensional location in the model) between AO and action-related language (in the first dimension on the x axis), between action execution (in the second and third dimensions on the y and z axes), and between AO-MI (in the second dimension on the y axis). Differing on at least one dimension suggests that each action type has some unique neurocognitive characteristic that contributes to behaviour. Notwithstanding this, most action types were also found to be similar (i.e., with small dissimilarity effect size) to all other action types on at least one neurocognitive dimension, the exception to this being action-related language (which had medium to large dissimilarity with all action types). Similarity across action types on at least one dimension is consistent with neurophysiological findings of overlapping brain regions (Caspers et al., 2010; Hétu et al., 2013; Hardwick et al., 2018). The finding of both unique and similar neurocognitive dimensions across action types assists in explaining why alternating or combining action types during skill learning or practice contributes to positive outcomes over and above those observed using a single action type (Larson and Suchy, 2015; Caligiore et al., 2017; Larssen et al., 2021; Scott et al., 2021; Bazzini et al., 2022). Overall, the 3-dimensional model shows that across all action types, action execution and concurrent AO-MI were most similar, as indicated by the minimal to small dissimilarity effect sizes on two of the three dimensions in the configuration (see Table 4). Additionally, the most dissimilar action types were action execution and AO, as indicated by the very large dissimilarity effect sizes in the first and third dimensions in the model configuration (Table 4). The following section will discuss specific findings in more detail.

The finding of significant dissimilarity between action execution and AO was predicted, and based on the consideration of AO as a perceptual-cognitive function (see Kim et al., 2017), with less neural activation in purely motor centres than other action types (e.g., Caspers et al., 2010; Hardwick et al., 2018), and the absent corticospinal excitability during AO (Meers et al., 2020). However, the extent of dissimilarity (i.e., across all three dimensions) is also somewhat surprising given that research demonstrates, for example, that using AO interventions during limb immobilisation can mitigate associated negative effects by preserving sensorimotor cortical excitability during the immobilised period (Bassolino et al., 2014) and safeguarding motor performance (De Marco et al., 2021). Additionally, AO is known to be effective in learning novel actions, particularly when these involve coordination or sequencing actions (Boutin et al., 2010; Gatti et al., 2013; Gonzalez-Rosa et al., 2015; Cuenca-Martínez et al., 2020). Indeed, AO has been shown to outperform MI in learning unfamiliar actions (Gatti et al., 2013; Gonzalez-Rosa et al., 2015). Further, when observing actions that are familiar to us, the content of mental representation during AO appears to include both visual and motoric components (Calvo-Merino et al., 2006). Consequently, it cannot be discounted in the present study that the considerable dissimilarity between AO and action execution may reflect the context in which AO was performed in the neurophysiological studies entered into the NMDS analysis. In this regard, only studies that used AO in the context of passive observation of action were included for analysis, due to the possibility that AO in the context of, for example, subsequent intention to imitate may encourage active motor processing or implicit MI. Furthermore, research indicates that the tasks and context associated with AO appear to modify the processes engaged during AO, in that, different strategies may be adopted, some of which are less motoric (or not motoric at all) than others (Hodges, 2017). It is worth noting that evidence suggests that AO activates motor centres (thus displaying neural overlap with action execution) to a greater extent when biological vs. non-biological movements are observed, or when movements are familiar rather than unfamiliar, and so, the importance of the context in which AO is performed appears to be an important consideration (Holz et al., 2008; Zentgraf et al., 2011).

It is interesting that AO was found to be most similar to MI over all other action types (as indicated by the small dissimilarity effects between AO and MI on two of the three dimensions; albeit having a significant large dissimilarity effect with MI on the first dimension). The similarity between AO and MI on two dimensions (i.e., dimensions 2 and 3 on the y and z axes, respectively) may reflect their mental status (i.e., their reliance on cognitive systems, such as memory, attention, etc.) or their role in the cognitive transformation of motor-related information *via* a simulation mechanism (Jeannerod, 2001, 2006). This idea is somewhat supported by the observation that on these same two dimensions MI and action execution, and AO and action execution, show dissimilarity, and so, the similarity between AO and MI (in conjunction with the dissimilarity between MI/AO and action execution) appears to relate more to cognitive aspects of action rather than motoric. The interconnection between AO and MI, and their mutual dissimilarity to action execution on two neurocognitive dimensions, supports previous research demonstrating that learning a motor skill *via* MI relies more on perceptual processes than does learning *via* action execution (Ingram et al., 2016).

Notwithstanding the similarity between AO and MI on two dimensions, it is important to note that MI and AO differ significantly on the first dimension (see Figure 2; Table 4). In this dimension, MI was most similar to action execution, as indicated by their proximity in the ordination model (and small dissimilarity effect sizes), which suggests that this first neurocognitive dimension may be motoric in nature (this is further discussed below). Given the extent of empirical evidence demonstrating positive effects of MI use on motor behaviour and neuroplastic change (Pascual-Leone et al., 1995; Debarnot et al., 2014; Di Rienzo et al., 2016), it was predicted that MI would be similar to action execution on at least one dimension, and this finding supports this.

Overall, it was expected that action execution would display some dissimilarity to all covert action types (i.e., MI, AO, action-related language), given the mental–physical gradient between them (Jeannerod, 2006; Glover and Baran, 2017). This prediction was largely satisfied as indicated by large dissimilarity effect sizes in at least two dimensions between action execution and each action type (an exception being AO-MI). As already stated, action execution was more dissimilar to AO than any other action type across the three dimensions. While execution was similar to MI on the first dimension, it is interesting that in the present study, action execution was most similar to concurrent AO-MI than any other action type, as indicated by the minimal to small dissimilarity effect sizes in location on two of the three dimensions in the configuration (see Table 4). Action execution involves both perceptual and predictive processes for control of movement in the environment (e.g., Wolpert and Ghahramani, 2000; Grush, 2004; Pezzulo et al., 2013), and according to the similarities observed in the NMDS model presented here (see Figure 1) it may be that combined AO-MI also largely capture these qualities. Research demonstrates that with walking actions, gait phase dependent modulation of cortical activity in sensorimotor areas (as measured by electroencephalography; EEG) during combined AO-MI, was more similar to that during execution of the same action than was AO alone (Berends et al., 2013; Kaneko et al., 2021). Further, concurrent AO-MI (during balance control) appears to activate neural motor centres (e.g., M1, PMC, SMA, and cerebellum) to a greater extent than either MI or AO alone (Taube et al., 2015), and also enhance corticospinal excitability (Sakamoto et al., 2009). When examining primary motor cortex (M1) activity, 9% of AO-related studies and 100% of action execution studies included in the NMDS analysis indicated activity in the M1. Additionally, 27% of MI studies and 40% of concurrent AO-MI studies included in the model displayed such activity. This example of neural motor activity patterns highlights a superadditive effect (in AO-MI), where using action types concurrently, or interspersed, appears to have greater influence on brain activity and/or movement performance than using either alone (e.g., Romano-Smith et al., 2018; Larssen et al., 2021; Bazzini et al., 2022; for review see Scott et al., 2021). The greater movement skill improvement typically observed when physical performance is interspersed either AO or MI may occur because somewhat different but complementary processes are refined during covert and overt action which ultimately leads to superior behaviour (Allami et al., 2008; Malouin et al., 2013; Larssen et al., 2021).

The first dimension displays similarity (i.e., small effect sizes in location difference in the ordination model) between action execution and MI and AO-MI, and so, it may be that this dimension can be characterised as motoric in nature. When the similarity between action execution and MI in the first dimension is interpreted in conjunction with the observation of large dissimilarities between AO and AO-MI and between AO and MI and small group differences in location between MI and AO-MI and between action execution and AO-MI, it appears that in AO-MI on this dimension it is MI that is primarily driving processing, thus supporting the previous research finding that MI during AO-MI drives corticospinal excitability (see Meers et al., 2020). The apparent motoric nature of MI in this dimension may relate to the predictive forward modeling mechanism of motor control theories (i.e., efference copy; Wolpert and Ghahramani, 2000) given the offline or cognitive status of MI. It is also interesting that the motoric quality of this dimension is further implied by the location of action-related language (assumed to be a motor-related but more cognitive process) which displays large group differences in proximity to the locations of MI, action execution, and AO-MI.

It was further predicted in the present study that action-related language will show some similarity to AO and action execution on at least one dimension. This prediction was based on the observation that previous research indicates that action-related words trigger activation of the motor system (Pulvermüller et al., 2005; Pulvermüller, 2005, 2018; Jirak et al., 2010; Courson and Tremblay, 2020) and that AO or action execution prime action-related language processing (Beauprez et al., 2020; Bidet-Ildei et al., 2020). The findings relating to the configuration of the NMDS model indicate that this prediction is not supported, as action-related language shows medium to large dissimilarity with all action types across the three dimensions (see Table 4). Given that previous research findings suggest functional links between sensorimotor systems and language systems (e.g., Pulvermüller et al., 2005; Larson and Suchy, 2015; Bonnet et al., 2022), it is possible that the action-related language tasks used in the studies entered into the model in the present study influenced the outcome. Specifically, although research demonstrates the role of sensorimotor brain areas in word recognition, there are indications that context, experience, or familiarity with action influence the strength of link between action and language (e.g., with motor experience, language processing is facilitated, for example, with faster times for word recognition; Pulvermüller et al., 2005; Holt and Beilock, 2006; Beauprez et al., 2019, 2020). Accordingly, context may determine the extent of involvement or mediation of sensorimotor systems in language processing (Arbib et al., 2014; Moreno et al., 2015). Facilitative effects of motor experience may be linked to the operation of associative mechanisms (e.g., Pulvermüller, 2005) or simulation mechanisms (Jeannerod, 2006; Barsalou, 2008), which elaborate action representational content over time, and could account for the positive effects of MI training on semantic access/categorisation tasks (Bonnet et al., 2022) or AO training on verb processing (Beauprez et al., 2020). Overall, it appears that the relational connections between action-related language and other action types is complex and will require further nuanced investigation to unravel the specificities of any links that may exist. For example, it will be useful to fully understand the temporal organisation of processing associated with each action type in conjunction with action-related language processing, as this may provide insight into whether action-related language is more related to cognitive dimensions than motor dimensions, and whether action words are understood and processed prior to, simultaneously with, or after activation of motor systems (e.g., Moreno et al., 2015; Zappa et al., 2019).

There are some methodological limitations in the present study. Firstly, the data for the study was based on data previously generated across different neuroimaging studies. One drawback of this is that different tasks and experimental requirements were involved which may have impacted the extent or type of brain activation recorded. Although this potential confound is mentioned here, the present analysis included 33 articles (out the total 53) that studied more than one action type using the same experimental conditions, and so the impact of such a confound is mitigated. Secondly, a single author searched the literature for inclusion of appropriate studies and reviewed the activated brain areas described, which may have led to missed or overlooked potentially viable articles or brain activations. However, a single reviewer ensured consistency across selection procedures and so has advantages. Finally, the number of cases of AO+MI entered into the model was only half that of the number of cases entered for other action types. Unfortunately, although this does not impact the NMDS solution calculation, it reflects the limited amount of neuroimaging research on this particular action combination.

The present study highlights both similarities and dissimilarities between four action types on three dimensions, which somewhat supports the idea of complementarity. However, given the potentially important implications of using action types in combination (either simultaneously or alternating) for movement skill improvement or rehabilitation, it is important that future research strives to fully understand how best to integrate different action types across different tasks, contexts, and disciplines. In this regard, recent attempts to integrate different action types into a single method for rehabilitation of language (e.g., Durand et al., 2021) or motor function in the clinical (e.g., Bek et al., 2021; for review see Caligiore et al., 2017) and sport (e.g., see Wright et al., 2021) domains represent important avenues.

## Conclusion

The primary aim of this study was to use non-metric multidimensional scaling to reduce the dimensions, and thus complexity, of the neurocognitive substrate of the four different action types of motor imagery (MI), action observation (AO), action execution, and action-related language, to identify the least number of dimensions that distinguish or relate them. Understanding their relational connections contributes to a better understanding of the neurocognitive mechanisms driving their functioning, and the extent to which these mechanisms might be shared by the different action types. Taking the results of the current study together, three main findings are reported. First, action execution and AO (in the first and third dimensions on the x and z axes, respectively) and MI and AO (in the first dimension on the x axis) showed significant dissimilarity, with action execution and AO displaying the most dissimilarity across all other action types. Second, in the three-dimensional model, across all action types, action execution and concurrent AO-MI were most similar. Finally, action-related language demonstrated the greatest dissimilarity with all action types across the three dimensions in the model. Overall, each action type showed some unique neurocognitive characteristics (with dissimilarity to all other action types on at least one dimension) that likely contributes to the optimal functioning of the perceptual-cognitive-motor system, allowing humans to perform, observe, learn, imagine, and reason about action-related information. The findings herein position four different action types in a shared three dimensional space that highlights the extent of their neurocognitive commonalities and dissimilarities. By doing so, it is anticipated that this will assist in understanding how best to exploit the various interconnections between these different covert actions in the rehabilitation of any one that is dysfunctional. Recent work exploiting the combination of AO and MI for application in clinical (e.g., Scott et al., 2021) and sport domains (see Wright et al., 2021), and the combination of action observation, execution, and imagery in assisting fluency in action-related language (e.g., Durand et al., 2021) are promising directions. As yet a single comprehensive theory of motor cognition that accounts for the accumulated evidence (and for different but overlapping hypotheses or theories) has yet to emerge. Future research might concentrate efforts on further cultivating and testing a comprehensive theory of action-related functions, with particular focus on whether they are best described and explained by motoric or non-motoric conceptualisations.

## Data Availability Statement

The original contributions presented in the study are included in the article, further inquiries can be directed to the corresponding author.

## Author Contributions

HO’S is the sole intellectual contributor to the work.

## Footnotes

^1^ https://pubmed.ncbi.nlm.nih.gov
